# Potential Risk Factors of Smokeless Tobacco Consumption Among Adolescents in South India

**DOI:** 10.1093/ntr/ntac003

**Published:** 2022-02-09

**Authors:** Muralidhar M Kulkarni, Veena G Kamath, Asha Kamath, Sarah Lewis, Ilze Bogdanovica, Manpreet Bains, Jo Cranwell, Andrew Fogarty, Monika Arora, Gaurang P Nazar, Kirthinath Ballal, Ashwath K Naik, Rohith Bhagawath, John Britton

**Affiliations:** Community Medicine, Kasturba Medical College, Manipal Academy of Higher Education, Manipal, Udupi, Karnataka, India; Community Medicine, Kasturba Medical College, Manipal Academy of Higher Education, Manipal, Udupi, Karnataka, India; Department of Data Science, Prasanna School of Public Health, Manipal Academy of Higher Education, Udupi, Karnataka, India; UK Centre for Tobacco and Alcohol Studies, University of Nottingham, Nottingham, UK; UK Centre for Tobacco and Alcohol Studies, University of Nottingham, Nottingham, UK; UK Centre for Tobacco and Alcohol Studies, University of Nottingham, Nottingham, UK; Department for Health, University of Bath, Bath, UK; UK Centre for Tobacco and Alcohol Studies, University of Nottingham, Nottingham, UK; HRIDAY, Delhi, India; Health Promotion Division, Public Health Foundation of India, Delhi, India; HRIDAY, Delhi, India; Health Promotion Division, Public Health Foundation of India, Delhi, India; Community Medicine, Kasturba Medical College, Manipal Academy of Higher Education, Manipal, Udupi, Karnataka, India; Department of Data Science, Prasanna School of Public Health, Manipal Academy of Higher Education, Udupi, Karnataka, India; Community Medicine, Kasturba Medical College, Manipal Academy of Higher Education, Manipal, Udupi, Karnataka, India; UK Centre for Tobacco and Alcohol Studies, University of Nottingham, Nottingham, UK

## Abstract

**Introduction:**

Although most of the disease burden internationally is due to tobacco smoking, smokeless tobacco (SLT) use contributed to an estimated 76 000 deaths in 2017. We have studied the potential risk factors for SLT use among adolescents in South India.

**Methods:**

A cross-sectional questionnaire survey of all students in grades 6–8 in schools in the Udupi district of Karnataka State ascertained SLT use status and potential determinants of SLT uptake. Ever SLT use was defined as any reported consumption of any SLT products, currently or at any time in the past. Independent effects on ever SLT use status were estimated using multiple logistic regression.

**Results:**

Of 46 706 students from 914 participating schools, 39 282 (84.1%) provided questionnaire responses sufficiently complete for analysis. Ever SLT use was reported by 775 (2.0%) participants and in a mutually adjusted model was significantly related to age, male sex, family use, or friend’s use of SLT, low socioeconomic status, high rebelliousness, and low self-esteem. After controlling for these effects, the odds of ever-SLT use were significantly higher among students who had least awareness of the harmful effects of tobacco use (odds ratio 3.7, 95% confidence interval [2.9, 4.7]) and significantly lower among those not exposed to tobacco advertising (odds ratio 0.7, 95% confidence interval [0.5, 0.8]).

**Conclusions:**

The prevalence of SLT use among children in Karnataka is relatively low when compared with other studies in India. The significant potential risk factors of SLT use include low awareness of the harmful effects of tobacco and tobacco control policies and exposure to tobacco advertising.

**Implications:**

The prevalence of SLT use among school going adolescents in South India is relatively low. The potential risk factors for SLT use among adolescents in southern India are similar to those for smoked tobacco. It includes age, male gender, family or friend’s use of SLT, low socioeconomic status, high rebelliousness, low self-esteem, exposure to tobacco advertisement and least awareness about the harmful effects of tobacco and of tobacco control policies. The present study lays emphasis regarding creating awareness about tobacco harms and control policies for further reducing tobacco use among adolescents.

## Introduction

Tobacco use is a major cause of global morbidity and mortality, currently accounting for over 8 million deaths each year.^1^ Most of this disease burden arises from tobacco smoking, but smokeless tobacco (SLT) use contributed an estimated 76 000 deaths in 2017.^2^ Globally, there are around 357 million adult SLT users, of whom 83% live in the Southeast Asian region, where SLT use has been reported to be more prevalent than tobacco smoking.^3^ The use of SLT has been reported in 127 countries in different continents across the world and particularly, in the Southeast Asian region^4^ where it is an accepted practice in society and widely perceived to be less harmful than smoked tobacco.^5^ They are also marketed by the tobacco industry for use as short-term substitutes for smoking in smoke-free environments.^5^

In terms of DALY’s lost, more than 85% of the SLT-related burden is in South and Southeast Asia, of which India accounted for 70%, Pakistan for 7%, and Bangladesh for 5%.^4^ In a large case–control analysis carried out in India, it was observed that SLT use was associated with increased risk of cancers, cardiovascular diseases, and stroke. The age, sex, education, and study area adjusted mortality odds ratio was 30% higher (relative risk [RR]: 1.3, 95% confidence interval: 1.2–1.4) in ever tobacco chewers compared with never chewers and caused 7.1% of deaths from all medical causes.^6^

In India, SLT is easily available, affordable, attractively packaged, and used by more than one fifth of the population aged 15 years and older, with initiation of use often occurring before 10 years of age.^7,8^ Studies in India and other countries have identified gender, educational level, family influence, wealth index (inverse association), urban–rural residence, socioeconomic status, and low tax as important determinants of tobacco use.^9–12^

The SLT industry has grown exponentially in the past two decades in the country, suggesting that many more people have started using the product.^7^ In response to the widespread use of both smoked and smokeless tobacco products and their harmful consequence, the Government of India has introduced tobacco control policies and programs that include health warnings covering 85% of the surface of tobacco packs, banning the sale of gutkha, issuing tobacco-free educational institution guidelines and increasing taxation.^8,13–15^ Among children, use of SLT in adolescents aged 13–15 years in India has declined from 14.0% in 2003 to 4.1% in 2019.^16^

The myriad ways of SLT use, its product and sale mainly being an unorganized sector, and limited research on risk factors of SLT use and enormous health impact associated with it warrant further research ^17^. Hence, this study was carried out in Udupi, a district in south India that is self-declared as high compliance district with respect to implementation of Cigarettes and Other Tobacco Products Act (COTPA), to identify the gaps that could help in further strengthening the tobacco control measures. This study, which is part of a larger research work on all forms of tobacco use, presents data on the potential risk factors of SLT use among school going adolescents.

## Methods

We used a cross-sectional questionnaire survey to measure the prevalence and determinants of SLT use in students in grades 6, 7, and 8 (aged between 10 and 15 years) in all the consenting schools of Udupi district in Karnataka State in Southern India which is a high compliance district^18^ for implementation of tobacco control policies. The said district was chosen as the findings drawn from survey among children living in an environment in which government tobacco control policy is already in place, will provide an insight into the effectiveness of and a potential for improvement in these policies. After obtaining the principal’s consent, researchers visited the school twice: first, to distribute a study information sheet for students and parental information and opt-out consent forms to all students in grades 6–8, and second, during a period of approximately 45 minutes scheduled into the schooldays, 3–14 days later, to distribute questionnaires for completion by all consenting students whose parents did not exercise the opt-out.

Of 924 schools with 6-8 grades in Udupi district, 914 schools agreed to be part of the survey. As school attendance rates are high, we studied only those children present on the arranged study day; if for any reason (e.g., heavy monsoon rains) fewer than 80% of students were in attendance, the survey was rescheduled. The surveys did not contain any personal identifiers, and confidentiality was maintained. Further details about survey method is provided in a paper on tobacco smoking in this population.^19^

### Questionnaire Design and Study Variables

The questionnaire elicited information on current and past use of SLT and frequency of use (never; ever but not now; less than once a week; once a week; daily) for products including chewable tobacco, gutka, khaini, zarda, and snuff,^20^ using questions adapted from the Global Youth Tobacco Survey^21^ and HRIDAY’s Mobilizing Youth for Tobacco-Related Initiatives in India (MYTRI) project.^22^ Questions on exposure to and awareness of tobacco products in retail outlets, including brand recognition, were adapted from those used for smoked tobacco in the Nottingham Schools study.^23,24^ The students were asked to mention the names of the tobacco brands if they had noticed during their visit to Point of Sale. Awareness of health warnings and mass media campaigns were evaluated using questions from the GYTS-India and Nottingham Schools surveys, adapted to include awareness of graphic and printed health warnings, and recall of exposure to tobacco control media campaigns. Questions were included on the tobacco control policies adopted by the respondent’s school and in the family home, and on family SLT use, peer SLT use, self-esteem, and rebelliousness.^25–27^ We measured socioeconomic status through a question on ownership of household goods, grouping participants into quintiles of family wealth.^28^ We also asked students how often they visit small shops or supermarkets and whether they notice tobacco brands on display during their visit, using previously validated questions^24^ with responses categorized as binary variables, indicating that they had noticed tobacco brands on display during their visit to the points of sale, or had not. The questions on awareness included the effect of tobacco use on health; harms of tobacco use; tobacco control policies such as laws banning sale of gutkha (a SLT product), tobacco-free school policies and regarding the ban on sale of tobacco products to minors. Correct responses were given a score of one and an incorrect response zero, summed for each student and grouped into tertiles representing levels of knowledge labeled as low, medium, and high. Other variables included are explained in detail in Supplementary File 1.

### Data Analysis

Data were extracted from completed questionnaires into Microsoft Excel using Optical Mark Reader scanning and transferred into STATA 9.2 software for analysis. Ever SLT use was defined as any reported SLT product use, currently or in the past and current SLT use as use of any SLT product in the last 30 days. Associations between ever SLT use and all variables were evaluated using logistic regression to estimate the effects of potential explanatory variables on the risk of SLT use. Demographic and environmental variables were explored first and all which were significantly (*p* < .05) associated with ever SLT use were retained in the model. The potential for collinearity (for example maternal and paternal education) was explored by fitting each individually in the model. We then included the variables measuring antitobacco and protobacco activities and awareness of tobacco hazards and tobacco control policies that were significantly associated in univariate analysis in the model. We used the logistic regression model instead of the log binomial model as the prevalence of ever SLT use was lower than 10%.

Ethics approval was granted by the Manipal and Nottingham University Ethics Committees, Centre for Chronic Disease Control, India and the Health Ministry’s Screening Committee. Data were collected between July 2017 and January 2018.

## Results

Of the 46 706 students in grades 6–8 in 914 schools, 3066 were absent on the day of the survey, 271 declined to participate, 315 were excluded by parental opt-out, and 6 students consented but did not complete a questionnaire. The remaining 43 048 students (92%) completed the survey questionnaire. After excluding 3766 questionnaires with insufficient or otherwise unusable data, 39 282 questionnaires (representing 84% of the eligible students in consenting schools) were available for analysis. Respondents included similar proportions of males (51%) and females (49%), and most participants were of Hindu religion (83.3%) and from rural areas (80.1%).

Ever SLT use was reported by 775 (2.0%) students, and in multivariate analysis differed significantly between ages, being most common among 11 year olds; and being significantly more common among males (1.7 [1.4, 2.0]), SLT use allowed at home (3.2 [2.7, 3.8]), respondents reporting family use or friend’s use of SLT, those of low socioeconomic status, high rebelliousness, low self-esteem and below average school performance (1.9 [1.3, 2.7]) (Supplementary Table 1).

Among SLT ever users, age at initiation was 10 or lower for 29.6% of students (Table 1). Similar proportions of students used each of the five different types of SLT that included snuff, khaini, chewable tobacco, gutkha, and zarda, and of those reporting frequency of use, almost half had used no more than one pack in the past 30 days (Table 1). Current use of SLT was reported among 381(49.2%) of ever users and amounts to 1.0% of the study population. Likewise, 412 (53.2%) of the ever users reported to have used smoking products also, with a prevalence of 1.0% among the study population.

**Table 1.** Characteristics of Smokeless Tobacco Initiation Among Ever Smokeless Tobacco Users (*n* = 775)

Characteristic	Number	%
Smokeless tobacco use (*n* = 39 282)		
Never used	38 507	98.0
Used smokeless tobacco in past	338	0.9
Used smokeless tobacco sometimes but less than once a week	200	0.5
Used smokeless tobacco one to six times in a week	120	0.3
Used smokeless tobacco more than six times a week	117	0.3
Current smokeless tobacco use (*n* = 775)		
Not used in last 30 days	392	50.6
Used in last 30 days	381	49.2
No response	2	0.2
Age at initiation (years) (*n* = 775)		
<7	44	5.7
8	67	8.7
9	21	2.7
10	98	12.7
11	49	6.3
12	97	12.5
13	28	3.6
14	39	5.0
15	66	8.5
No response	266	34.3
Type of tobacco use (*n* = 775)		
Smokeless tobacco users	363	46.8
Both smoking and smokeless (dual) tobacco users	412	53.2
Products used in last 30 days		
Snuff	54	7.0
Khaini	77	9.9
Chewable tobacco	62	8.0
Gutkha	67	8.7
Zarda	59	7.6
Others	62	8.0
No response	394	50.8
Packets used in past 30 days		
I used but did not buy	47	6.1
1 packet	146	18.8
2–5 packets	56	7.2
5–10 packets	76	9.8
>10 packets	54	7.0
No response	396	51.1

After adjusting for the effects of independent significant variables identified in Supplementary Table 1, use of SLT was found to be significantly less common among those who had not participated in antitobacco activities or were aware of the health harms of tobacco or current tobacco control policies or who had not seen tobacco advertising (Table 2). There was no significant association between SLT use and participating in an educational class on health hazards of tobacco and having heard or seen antitobacco media messages (Table 2). Having seen tobacco products at the point of sale in shops was not associated with SLT use on univariate analysis and hence it was not included in the model for analysis.

**Table 2.** Exposure to Antitobacco and Protobacco Activities and Ever Smokeless Tobacco Use

Characteristic	Number	Ever users (%)	Crude OR (95% CI)	p	Adjusted OR** (95% CI)	p
Class on health hazards of tobacco				<.001		.089
Yes	7917	208 (2.6)	1		1	
No	31 365	567 (1.8)	0.7 (0.6, 0.8)		0.8 (0.7, 1.0)	
Participated in antitobacco activity				<.001		<.001
Yes	7675	276 (3.6)	1		1	
No	31 607	499 (1.6)	0.4 (0.4, 0.5)		0.5 (0.4, 0.6)	
Heard or seen antitobacco media messages				<.001		.252
Yes	25 176	572 (2.3)	1		1	
No	14 106	203 (1.4)	0.6 (0.5, 0.7)		0.9 (0.7, 1.1)	
Awareness of tobacco harms and control policies (grade)				<.001#		<.001#
<6 (low)	9057	394 (4.4)	5.3 (4.2, 6.5)		3.7 (2.9, 4.7)	
6–7 (medium)	17 877	275 (1.5)	1.8 (1.4, 2.3)		1.8 (1.4, 2.3)	
>7 (high)	12 348	106 (0.9)	1		1	
Exposure to tobacco advertisements				<.001		<.001
Yes	30 111	668 (2.2)	1		1	
No	9171	107 (1.2)	0.5 (0.4, 0.6)		0.7 (0.5, 0.8)	
Exposure to tobacco products display at PoS				.338		
Yes	36 196	707 (2.0)	0.9 (0.7, 1.1)			
No	3086	68 (2.2)	1			

CI = confidence interval; OR = odds ratio.

^**^Adjusted OR are mutually adjusted for all significant variables in univariate analysis in Table 2 and significant variables of multivariate model of Supplementary Table 1 that included age, gender, home smokeless tobacco use allowed, family and Friend Smokeless tobacco use, wealth quintile, rebelliousness, self-esteem, school performance.

^#^ *p* value for trend.

## Discussion

This study shows that SLT use among adolescents in grades 6–8 in schools in southern India was uncommon, at 2%, but more common among males, those of lower socioeconomic status, homes where SLT use is allowed, those whose friends and family used SLT, and among the more rebellious and those with lower self-esteem. SLT use was more common among those with low awareness of the health harms of tobacco and tobacco control policies, those who had participated in antitobacco activities, and those who reported exposure to tobacco advertising.

The potential risk factors of SLT use observed in our study are similar to those found in other studies done not only in India but also from other Southeast Asian countries.^7,10,29^ Although our study was done in a single district, our sample included all schools in rural, urban, and semiurban localities of the district and achieved a very high participation rate in a relatively short time interval. Our findings are thus likely to be highly representative, though the cross-sectional study design inevitably limits any inference as to the causal nature of the risk associations identified. The overall prevalence of SLT use in our study population was low in relation to those from other countries in Southeast Asia region and in southern India, but our study population was younger than those in previous reports.^5,30,31^ On these grounds, the expected finding that SLT use was more common among males similar to global estimates^31^ suggests that our findings are likely to be representative of the wider population of children in southern India.

In previous studies in India, the prevalence of SLT use has typically been higher than that of smoking,^20,30^ but in the present study, the prevalence of SLT use was lower, at 2%, than that of smoking (2.4%) in this cohort.^19^ Whether this contrast represents a declining trend in SLT use is not clear. It is also not clear whether the lower prevalence of SLT use signifies diversion of adolescents from use of smokeless to smoked tobacco. The extent to which these differences are due to the types of SLT available or local or national policies on tobacco use is unknown.

Among adults, the GATS II survey in 2016–2017 for India has revealed a relative reduction of 33% in SLT use compared with the GATS I survey in 2009–2010 among people aged 15 years and older.^32^ Similarly, among adolescents aged 13–15 years also SLT use has reduced by 50% between 2009 and 2019 as per GYTS, surveys III^8^ and IV,^16^ respectively.

Surveys on age at initiation of SLT use in different parts of the country have reported varying proportions of children initiating SLT use by the age of 10 years, with variation from 30% in North India to 65% in North East India.^33,34^ In another study done in a neighboring state to Karnataka, 40% of children in schools had tried tobacco products by age of 10.^35^ Although our study was done a decade later and a relatively high proportion of participants did not provide data on the type or quantity of SLT used, still 29.6% of ever users reported to have tried SLT products by the age of 10. Besides, in recently released GYTS IV report (2019), the median age of starting SLT use is 9.9 years in India. This observation highlights the need to develop interventions to try to prevent this uptake of tobacco at a young age.

The commonly used SLT products in our study included Khaini (nearly 10%) and gutkha (nearly 9%) and is consistent with adult use pattern seen in GATS.^32^ Furthermore, it also indicates that despite the ban (in 2013 in Karnataka),^36,37^ Gutkha is still available and accessible even to minors. This requires an enhanced enforcement of Gutkha ban in the state. Although exposure to tobacco products display at point of sale (PoS) did not have significant association with SLT use, studies done in United Kingdom reported PoS display to increase susceptibility to smoking. This contrasting findings could be due to difference in the existing policies and its implementation in the two countries.^23^

Our study demonstrates that adolescents who are relatively aware of the health hazards of tobacco use or tobacco control policies are less likely to use SLT, a finding in line with previously reported evidence^5,7^ though merely attending classes on health hazards was not found to be significantly associated with SLT use in contrast to other studies.^11^ This emphasizes the importance of educating adolescents about tobacco-related harms and tobacco control policies in this vulnerable age group as a means to prevent initiation of tobacco use. However, we also observed that participating in antitobacco activities was associated with a higher likelihood of SLT use similar to findings from a secondary data analysis of South Asian adolescents,^29^ and while the explanation for these conflicting findings is not clear, one possibility is that the former is a measure of understanding of harms, while the latter represents mere participation in, but not necessarily understanding of, tobacco health messaging. Another possible interpretation is that children who are participating in antitobacco activity may choose SLT over smoking because they understand that SLT use, while harmful, is far less harmful than smoking.

This study thus demonstrates that SLT use among adolescents in Karnataka is less compared to the national average, but remains a potential health problem and that further investment in effective antitobacco messaging is likely to bring down the prevalence of SLT use. It would be appropriate to strengthen the on-going school-based awareness sessions under the National Tobacco Control program and enforce tobacco-free educational institution guidelines in order to reach out to a larger number of adolescents and influence behavior change at a young age. There is a need for further research to develop a comprehensive module such as specific teaching topic on preventing tobacco uptake for adolescents in school settings. This needs to be supplemented by regular monitoring of the tobacco use among school children and staff along with evaluation of the existing policies and programs to avert rapid increase in prevalence of SLT use in later years.

## Supplementary Material

A Contributorship Form detailing each author’s specific involvement with this content, as well as any supplementary data, are available online at https://academic.oup.com/ntr.

ntac003_suppl_Supplementary_Table_S1

ntac003_suppl_Supplementary_Data

ntac003_suppl_Supplementary_Taxonomy-form
