# Supplementary material for: Potential Risk Factors of Smokeless Tobacco Consumption Among Adolescents in South India
Source: Nicotine Tob Res. 2022 Feb 9;24(7):1104–9. doi: 10.1093/ntr/ntac003 (PMC9199931; doi:10.1093/ntr/ntac003)
Supplement: ntac003_suppl_Supplementary_Table_S1 [file ntac003_suppl_supplementary_table_s1.docx]

**Supplementary Table 1. Demographic and environmental associations with Smokeless tobacco use in the study population, with univariate and mutually adjusted odds ratios.**

| **Characteristic** | **Number** | **Ever users (%)** | **Crude OR (95% CI)** | **P value** | **Adjusted OR ***  **(95% CI)** | **P value** |
| --- | --- | --- | --- | --- | --- | --- |
| **Age** |  |  |  | <0.001 |  | <0.001 |
| 10 | 217 | 7(3.2) | 1 |  | 1 |  |
| 11 | 5760 | 161(2.8) | 0.9(0.4,1.9) |  | 1.2(0.5,3.0) |  |
| 12 | 12932 | 297(2.3) | 0.7(0.3,1.5) |  | 1.0(0.4,2.6) |  |
| 13 | 13247 | 192(1.4) | 0.4(0.2,0.9) |  | 0.7(0.3,1.8) |  |
| 14 | 6671 | 107(1.6) | 0.5(0.2,1.1) |  | 0.7(0.3,1.8) |  |
| 15 | 455 | 11(2.4) | 0.7(0.3,2.0) |  | 0.6(0.2,2.0) |  |
| **Gender** |  |  |  | <0.001 |  | <0.001 |
| Male | 20020 | 537(2.7) | 2.2(1.9,2.6) |  | 1.7(1.4,2.0) |  |
| Female | 19262 | 238(1.2) | 1 |  | 1 |  |
| **School locality** |  |  |  | 0.239 |  |  |
| Urban | 7803 | 141(1.8) | 0.9(0.7,1.1) |  |  |  |
| Rural | 31479 | 634(2.0) | 1 |  |  |  |
| **School type** |  |  |  | 0.195 |  |  |
| Govt. | 16786 | 337(2.0) | 1.1(0.9,1.3) |  |  |  |
| Aided | 7584 | 165(2.2) | 1.2 (1.0,1.5) |  |  |  |
| Private | 14912 | 273(1.8) | 1 |  |  |  |
| **Religion** |  |  |  | 0.004 |  | 0.529 |
| Christian | 2016 | 48(2.4) | 1 |  | 1 |  |
| Hindu | 32713 | 621(1.9) | 0.8(0.6, 1.1) |  | 0.9(0.6,1.3) |  |
| Jain | 152 | 5(3.3) | 1.4(0.5, 3.6) |  | 1.2(0.4,3.6) |  |
| Muslim | 4272 | 93(2.2) | 0.9(0.6, 1.3) |  | 1.0(0.6,1.4) |  |
| Other | 129 | 8(6.2) | 2.7(1.3, 5.9) |  | 1.7(0.7,4.3) |  |
| **Home Smokeless tobacco use allowed** |  |  |  | <0.001 |  | <0.001 |
| No | 35564 | 479 (1.3%) | 1 |  | 1 |  |
| Yes | 3718 | 296(8.0%) | 6.3(5.4,7.4) |  | 3.2(2.7,3.8) |  |
| **Family Smokeless tobacco use** |  |  |  |  |  |  |
| **Father** Yes | 2827 | 189(6.7%) | 4.4(3.7,5.2) | <0.001 | 2.6(2.1,3.2) | <0.001 |
| No | 36455 | 586 (1.6%) | 1 |  | 1 |  |
| **Mother** Yes | 354 | 65(18.4%) | 12.1(9.2,16.0) | <0.001 | 5.7(4.0,8.3) | <0.001 |
| No | 38928 | 710(1.8%) | 1 |  | 1 |  |
| **Siblings** Yes | 401 | 76(19.0%) | 12.8(9.8,16.6) | <0.001 | 3.5(2.5,5.0) | <0.001 |
| No | 38881 | 699(1.8%) | 1 |  | 1 |  |
| **others** Yes | 4214 | 118(2.8%) | 1.5(1.2,1.8) | <0.001 | 1.4(1.1,1.8) | 0.005 |
| No | 35068 | 657(1.9%) | 1 |  | 1 |  |
| **Friends Smokeless tobacco use** |  |  |  | <0.001 |  | <0.001 |
| None | 36311 | 323(0.9%) | 1 |  | 1 |  |
| One | 508 | 100(19.7%) | 27.3(21.4, 34.9) |  | 12.4(9.4,16.5) |  |
| Two | 355 | 134(37.7%) | 67.6(53.1,86.0) |  | 27.1(20.4,35.9) |  |
| Three | 356 | 96(27.0%) | 41.1(31.8,53.3) |  | 17.0(12.6,23.1) |  |
| Not sure | 1752 | 122(7.0%) | 8.3(6.7,10.3) |  | 5.2(4.2,6.6) |  |
| **Mothers education** |  |  |  | <0.001 |  | 0.153 |
| Illiterate | 2457 | 70(2.8%) | 2.2(1.4,3.7) |  | 1.2(0.6,2.1) |  |
| Primary | 11200 | 239(2.1%) | 1.7(1.1,2.6) |  | 1.2(0.7,2.1) |  |
| High school | 14466 | 282(1.9%) | 1.5(1.0,2.4) |  | 1.3(0.8,2.2) |  |
| Graduate | 3964 | 41(1.0%) | 0.8(0.5,1.4) |  | 0.8(0.5,1.5) |  |
| Postgraduate and above | 1554 | 20(1.3%) | 1 |  | 1 |  |
| **Fathers education** |  |  |  | <0.001 |  | 0.151 |
| Illiterate | 1757 | 56(3.2%) | 3.3(2.0,5.4) |  | 1.4(0.8,2.5) |  |
| Primary | 10008 | 196(2.0%) | 2.0(1.3,3.1) |  | 1.2(0.7,2.0) |  |
| High school | 14535 | 278(1.9%) | 1.9(1.2,3.0) |  | 1.5(0.9,2.5) |  |
| Graduate | 4129 | 66(1.6%) | 1.6(1.0,2.6) |  | 1.6(0.9,2.7) |  |
| Postgraduate and above | 2106 | 21(1.0%) | 1 |  | 1 |  |
| **Wealth Quintile** |  |  |  | <0.001 |  | <0.001 |
| Lower | 7735 | 277(3.6%) | 2.7(2.2,3.4) |  | 1.9(1.5,2.5) |  |
| Lower middle | 7315 | 150(2.1%) | 1.5(1.2,2.0) |  | 1.3(1.0,1.7) |  |
| Middle | 8464 | 153(1.8%) | 1.3(1.1,1.7) |  | 1.4(1.1,1.8) |  |
| Upper Middle | 7855 | 86(1.1%) | 0.8(0.6,1.1) |  | 0.9(0.7,1.2) |  |
| Upper | 7867 | 106(1.3%) | 1 |  | 1 |  |
| **Rebelliousness** |  |  |  | <0.001 |  | <0.001 |
| No | 24500 | 202(0.8%) | 1 |  | 1 |  |
| Mild | 10917 | 230(2.1%) | 2.6(2.1,3.1) |  | 2.2(1.8,2.7) |  |
| Moderate | 3396 | 272(8.0%) | 10.5(8.7,12.6) |  | 6.0(4.9,7.4) |  |
| Severe | 469 | 71(15.1%) | 21.5(16.1,28.6) |  | 8.0(5.5,11.5) |  |
| **High Self esteem** |  |  |  | <0.001 |  | 0.001 |
| Agree | 26968 | 435(1.6%) | 1 |  | 1 |  |
| Neither agree or disagree | 6817 | 179(2.6%) | 1.7(1.4,2.0) |  | 1.3(1.0,1.6) |  |
| Disagree | 5497 | 161(2.9%) | 1.8(1.5,2.2) |  | 1.5(1.2,1.8) |  |
| **School Performance** |  |  |  | <0.001 |  | <0.001 |
| Excellent | 15046 | 219(1.5%) | 1 |  | 1 |  |
| Good | 17993 | 275(1.5%) | 1.0(0.9,1.3) |  | 0.8(0.7,1.0) |  |
| Average | 5337 | 221(4.1%) | 2.9(2.4,3.5) |  | 1.4(1.1,1.8) |  |
| Below Average | 906 | 60(6.6%) | 4.8(3.6,6.4) |  | 1.9(1.3,2.7) |  |

*All p<0.05 after adjustment for age, gender, religion, family and friends SLT use, wealth quintile, rebelliousness, self-esteem and school performance.
