# Supplementary material for: Potential Risk Factors of Smokeless Tobacco Consumption Among Adolescents in South India
Source: Nicotine Tob Res. 2022 Feb 9;24(7):1104–9. doi: 10.1093/ntr/ntac003 (PMC9199931; doi:10.1093/ntr/ntac003)
Supplement: ntac003_suppl_Supplementary_Data [file ntac003_suppl_supplementary_data.docx]

**Key study variables:**

**Dependent variable:**

*Ever smokeless Tobacco use:* The question on tobacco use frequency comprised of options as never; ever but not now; less than once a week; once a week and daily. Ever SLT use was defined as any reported use of any smokeless tobacco product, currently or in the past. Thus any students marking any one of the last four options was considered as an ever-smoker.

*Current smokeless tobacco use*: The question on tobacco use frequency comprised of options as never, snuff, Khanini, Chewable tobacco Gutkha, Zarda and Others. Current tobacco use was defined as reported use of any smokeless tobacco product in last 30 days.

**Independent variables:**

Educational class on health hazards of tobacco; Participation in anti-tobacco activities; Heard or seen anti-tobacco messages on radio/TV, posters, newspapers / magazines; Observed tobacco advertisements on public transport vehicles, public places, internet, radio/TV, Newspapers/ Magazines; Exposure to tobacco products display at PoS.

*Educational class on health hazards of tobacco:* The student was asked whether he/she had a class on educational class on health hazards of tobacco in the past one year. The options included yes; no; and not sure.

*Participation in anti-tobacco activities:* We asked whether a student was involved in any anti-tobacco activity in the past one year.

*Anti-tobacco messages:* The questions were asked separately whether a student has heard or seen anti-tobacco messages on (a) radio/ TV (b) posters (c) Newspapers/ Magazines. The options included none, one to five messages, six to ten messages and more than ten messages. The response from these questions were combined to ascertain the exposure to anti-tobacco messages.

*Tobacco advertisements:* The students had to answer questions if they have noticed tobacco advertisements on public transport vehicles, public places, internet, radio/TV and Newspapers/ Magazines separately. The options were yes or no. The responses were then combined to assess whether a student was exposed to tobacco advertisement.

Exposure to tobacco products display at PoS : The students had to answer questions if they have noticed tobacco products on display when they went to super market and/ or small shops. And if they had noticed, they were asked to mention the name of the brand. All the students who mentioned name of any one of the tobacco brand were considered as exposed to display of tobacco products.

Knowledge about harmful effects of tobacco and tobacco control policies: The questions on awareness included the effect of tobacco use on health; harms of tobacco use; tobacco control policies such as laws banning sale of gutkha (a SLT product), tobacco-free school policies and regarding the ban on sale of tobacco products to minors. Correct responses were given a score of one and an incorrect response zero, summed for each student and grouped into tertiles representing levels of knowledge labelled as Low, Medium and High.

**Control variables:**

Age, gender, religion, school type, school locality, Fathers education, mothers education, Home smoking allowed, family members smoking, friends smoking, Wealth quintile, rebelliousness, self-esteem and school performance.

*School type:* Schools in Indian context are usually classified based on the source of funding and classified as Government, if the funding is by the state; Aided, when the funding is by the state but managed by a private agency; Private, if the school is self-funded without any funding by the state.

*School locality:* This was classified as urban or rural as per the Census 2011 guidelines.

Wealth quintile: This was assessed by grading them according to the facilities/ items present in their house.

*Rebelliousness:* We asked children a set of three questions based on a previous study conducted in SEAR countries. The questions included whether they ignore rules, - do things their parents wouldn’t want them to do, - get into trouble with authorities at school work or other places. The students had to mark as never, sometimes or often for these questions that were scored as 0, 1, and 2 and an aggregate score generated to classify children as having No, Mild, moderate and severe rebelliousness.

*Self-esteem:* This was assessed by a single item questionnaire based on whether they think they have high self-esteem and were asked to respond on a five point Likert scale which included options from strongly agree to strongly disagree.

*Age, gender, religion, Fathers education, Mothers education, family and friends smoking:* These responses were elicited by providing relevant options and children had to mark the most appropriate option.
